# Kidney Outcomes and Trajectories of Tubular Injury and Function in Critically Ill Persons with and without Coronavirus-2019

**DOI:** 10.21203/rs.3.rs-3974635/v1

**Published:** 2024-02-28

**Authors:** Michael L. Granda, Frances Tian, Leila R. Zelnick, Pavan K. Bhatraju, Mark M. Wurfel, Andrew Hoofnagle, Eric Morrell, Bryan Kestenbaum

**Affiliations:** University of Washington, Kidney Research Institute; University of Washington, Kidney Research Institute; University of Washington, Kidney Research Institute; University of Washington, Department of Medicine, Division of Pulmonary, Critical Care, and Sleep Medicine; University of Washington, Department of Medicine, Division of Pulmonary, Critical Care, and Sleep Medicine; University of Washington, Kidney Research Institute; University of Washington, Department of Medicine, Division of Pulmonary, Critical Care, and Sleep Medicine; University of Washington, Kidney Research Institute

**Keywords:** Coronavirus 2019, COVID, COVID-19, kidney, tubules, acute kidney injury, tubular secretion, tubular injury, tubular secretion, kim-1, egf

## Abstract

**Background:**

Coronavirus disease-2019 (COVID-19) may injure the kidney tubules via activation of inflammatory host responses and/or direct viral infiltration. Most studies of kidney injury in COVID-19 lacked contemporaneous controls or measured kidney biomarkers at a single time point. To better understand mechanisms of AKI in COVID-19, we compared kidney outcomes and trajectories of tubular injury, viability, and function in prospectively enrolled critically ill adults with and without COVID-19.

**Methods:**

The COVID-19 Host Response and Outcomes (CHROME) study prospectively enrolled patients admitted to intensive care units in Washington state with symptoms of lower respiratory tract infection, determining COVID-19 status by nucleic acid amplification on arrival. We evaluated major adverse kidney events (MAKE) defined as a doubling of serum creatinine, kidney replacement therapy, or death, in 330 patients after inverse probability weighting. In the 181 patients with available biosamples, we determined trajectories of urine kidney injury molecule-1 (KIM-1) and epithelial growth factor (EGF), and urine:plasma ratios of endogenous markers of tubular secretory clearance.

**Results:**

At ICU admission, mean age was 55±16 years; 45% required mechanical ventilation; and mean serum creatinine concentration was 1.1 mg/dL. COVID-19 was associated with a 70% greater incidence of MAKE (95% CI 1.05, 2.74) and a 741% greater incidence of KRT (95% CI 1.69, 32.41). The biomarker cohort had a median of three follow-up measurements. Urine EGF, secretory clearance ratios, and eGFR increased over time in the COVID-19 negative group but remained unchanged in the COVID-19 positive group. In contrast, urine KIM-1 concentrations did not significantly change over the course of the study in either group.

**Conclusions:**

Among critically ill adults, COVID-19 is associated with a more protracted course of proximal tubular dysfunction.

## INTRODUCTION

Coronavirus disease-2019 (COVID-19) is a viral syndrome caused by the severe acute respiratory syndrome coronavirus 2 (SARS-CoV-2). Clinical manifestations range from mild upper respiratory illness to acute respiratory distress syndrome (ARDS), multi-organ system failure, and death.(1, 2) Evidence suggests that SARS-CoV-2 infection may cause injury to the kidney tubules, either via direct viral infiltration and/or secondary activation of inflammatory host responses. In cell culture, SARS-CoV-2 directly infects proximal tubular cells, endothelial cells, and podocytes via the angiotensin-converting enzyme 2 (ACE2) receptor.(3, 4) Relatively high incidences of acute kidney injury (AKI) and kidney replacement therapy (KRT) are reported among hospitalized persons with COVID-19,(5–8) and markers of tubular injury such as kidney injury molecule-1 (KIM-1) are elevated early in the course of disease.(6, 9) Moreover, case series have described a syndrome of proximal tubular dysfunction among some patients with COVID-19 based on impaired reabsorption of beta-2-microglobulin, glucose, and uric acid.(10, 11)

On the other hand, most previous human studies of COVID-19 have lacked contemporaneously enrolled control persons without SARS-CoV-2, conflating potential kidney effects of this infection with the underlying severity of illness and temporal differences in care. Detectable SARS-CoV-2 is relatively uncommon in the blood(12) or urine(13) of patients with COVID-19, challenging the clinical relevance of direct kidney infection observed in cell culture. Yet, the mechanisms and natural course of injury to the proximal tubules remain poorly understood.

To that end, we sought to better define the patterns and longitudinal changes to the proximal tubules attributable to COVID-19 infection in critically ill patients. In this study, we compared the incidence of AKI outcomes and the trajectories of tubular injury, viability, and function in prospectively enrolled and comparably ill patients from intensive care units with and without COVID-19.

## METHODS

### Study population

The COVID-19 Host Response and Outcomes (CHROME) study prospectively enrolled 380 critically ill adults from intensive care units (ICU) at the University of Washington Medical Center, Harborview Medical Center, and Northwest Hospital, all in Seattle, WA, between April 2020 and May 2021.(14) Enrollment criteria were age ≥18 years, fever, hypoxemia (defined as requiring any supplemental oxygen or an oxygen saturation of <94% on ambient air), and symptoms of lower respiratory tract infection that prompted SARS-CoV-2 testing. Subsequent COVID-19 status was defined based on the results of rapid nucleic acid amplification testing (NAAT) of nasopharyngeal swabs, which were performed within 24 hours of ICU admission. The prospective enrollment of critically ill patients based on clinical suspicion for COVID-19 was designed to yield comparably ill cohorts of patients with and without the disease and minimize temporal differences in care. The CHROME study excluded persons who were pregnant, transferred from another ICU after more than 24 hours, had a history of solid organ transplantation, were institutionalized, or were unlikely to survive for more than 24 hours.

For the present study, we excluded23 CHROME patients who had a history of end-stage kidney disease (ESKD), six who had received dialysis prior to study enrollment, 20 with an admission serum creatinine concentration corresponding to an estimated glomerular filtration rate (GFR) <15 ml/min/1.73m^2^, and one without a collected urine sample, leaving 223 SARS-CoV-2 positive and 107 negative patients for analyses (“Clinical cohort”). We then measured biomarkers of kidney injury, viability, and secretory clearance in a subsample of 117 SARS-CoV-2 positive and 64 negative patients who had at least one paired plasma and urine sample for analysis (“Biomarker cohort”).

### Ethical Statement

Study procedures were approved by the Institutional Review Board (IRB), with consent obtained from all patients or waived by the local regulatory board early in the pandemic. All procedures were followed in accordance with the ethical standards of the responsible committee on human experimentation and with the Helsinki Declaration of 1975.

### Measurements of clinical study data

Study coordinators prospectively abstracted demographic data, respiratory status, vital signs, laboratory results, and the receipt of kidney replacement therapy (KRT) from electronic medical records. We calculated baseline Acute Physiology and Chronic Health Evaluation (APACHE) III and Sequential Organ Failure Assessment (SOFA) scores based on available clinical and laboratory data.(15) We determined the presence of acute respiratory distress syndrome (ARDS) based on the ratio of inspired to arterial oxygen concentration and adjudication of chest radiographs by an attending radiologist.(16)

Kidney outcomes were assessed over the course of hospitalization and included (1) the major adverse kidney event (MAKE), defined by at least a doubling of the serum creatinine concentration from baseline, requirement for kidney replacement therapy, or death,(17) (2) individual components of the MAKE outcome, and (3) any stage of acute kidney injury (AKI), defined by the Kidney Disease Improving Global Outcomes (KDIGO) as an absolute increase of ≥0.3 mg/dL or a ≥50% increase in serum creatinine from baseline.(18) We defined the baseline serum creatinine concentration as the clinically obtained value closest to, and before, the time of study enrollment on ICU day one. For 1 patient who did not have a serum creatinine measurement before ICU day 1, we used the first available clinical value after day one within 24-hours.

### Measurement of kidney biomarkers

Study coordinators collected blood and spot urine samples within 24 hours of ICU admission (day 1) and then subsequently on hospital days 3, 7, 10, and 14 if the patient remained hospitalized. Blood and urine samples were centrifuged at 3,000 RPM for 10 minutes at room temperature. We measured urine concentrations of kidney injury molecule 1 (KIM-1) and epithelial growth factor (EGF) using commercially available immunoassays (Enzo life sciences and R&D systems, respectively). The inter-assay variability is 6.2% for urine KIM-1 and 5.5% for urine EGF. We indexed measurements of KIM-1 and EGF to urine creatinine to account for variation in urinary concentration. We measured plasma concentrations of creatinine and cystatin C, and urine concentrations of creatinine and albumin using the Beckman-Coulter DxC Unicell 600. We estimated GFR in the biomarker cohort using the 2021 combined CKD-EPI equation based on plasma concentrations of creatinine and cystatin C.(19)

We estimated tubular secretory clearance based on plasma and urine concentrations of endogenous secretory solutes using a targeted liquid chromatography/mass spectrometry assay as previously described.(20) Plasma samples were precipitated in organic solvent and extracted using solid-phase extraction; urine samples underwent two parallel solid-phase extractions. Dried extracts were reconstituted in 80μl 5% acetonitrile/0.2% formic acid in H_2_O and passed through a large-pore filter plate (MSBVN1210; Millipore). Labeled internal standards were used to reduce sample-specific matrix effects and single-point external calibration was used to determine concentrations and reduce between-batch variability. We calculated the urine-to-plasma ratio for each solute as an approximation of their secretory clearance.(21) To facilitate interpretation and provide a single metric of secretory clearance, we also created a summary score by first standardizing each secretory ratio to a 0–100 scale:(20)

$$\text{Standardized rati}o_{X}=[ln(Ux/Px)-\text{min}(ln(Ux/Px))]/\text{range}(ln(Ux/Px))$$

where *ln(Ux/Px)* represents the log-transformed urine to plasma ratio of each solute, *min(ln(Ux/Px))* represents the minimal value in the distribution, and *range(ln(Ux/Px))* represents the range of these measurements. We then computed the summary score as the mean of the eight standardized ratios.

### Analytic plan

We tabulated baseline characteristics according to COVID-19 status using means and standard deviations for normally distributed data or medians and interquartile ranges for variables with skewed distributions. To increase the degree of similarity between SARS-CoV-2 positive and negative patients, we created a propensity score for SARS-CoV-2 positivity using logistic regression with the following clinical data at baseline: age, race, sex, body mass index (BMI), APACHE III score, SOFA score, admission source, extracorporeal membrane oxygenation, sepsis, trauma, pneumonia, history of hypertension, heart failure, chronic obstructive pulmonary disease, cancer, and diabetes, and use of beta blockers and diuretics. To assess covariate balance after weighting, we calculated weighted means and standard deviations (for continuous variables), and weighted proportions (for categorical variables) and then compared the standardized difference between covariates. Standardized differences below 0.25 are generally considered to indicate appropriate matching.(22)

For the MAKE outcome, patients were followed from the time of ICU admission until they either incurred a component of MAKE or their data was censored at hospital discharge. For outcomes of AKI, doubling of serum creatinine, and KRT, patients were censored for in-hospital death. We used weighted log-linear Poisson regression with robust Huber-White standard errors to estimate associations of baseline COVID-19 status with each clinical outcome. Models were weighted by the inverse probability of the COVID-19 propensity-score and additionally adjusted for the baseline serum creatinine concentration to control for confounding.

To model the trajectories of biomarkers over the course of hospitalization, we employed weighted generalized estimating equations with an independent working covariance structure to account for the correlation within person.(23) To account for selection bias that may arise from informative censoring, for each post-baseline sample collection, we constructed inverse probability of censoring weights (IPCW), by modeling the probability that the sample collection occurred with logistic regression, as a function of COVID-19 status and baseline covariates, including baseline measures of kidney function. At each time point, weights were the product of the baseline IPTW weights, divided by the probability of sample collections at the current and prior time points (i.e., IPCW). Within each group we estimated the mean daily change in kidney biomarkers using the slope after linear regression.

## RESULTS

### Baseline characteristics of the clinical study cohort

The clinical study cohort included 223 COVID-19 patients and 107 SARS-CoV-2 negative control patients (Table 1). The mean age at ICU admission was 55 16 years; 45% required mechanical ventilation; and 35% required vasopressors. The mean admission serum creatinine concentration was 1.1 mg/dL in each group. After propensity matching, baseline characteristics of COVID-19 patients and control patients were similar, including severity of illness scores and baseline serum creatinine concentrations. Baseline medication use was similar after propensity scoring (**Supplemental Table 2a**).

### Clinical kidney outcomes

**Figure 1:**
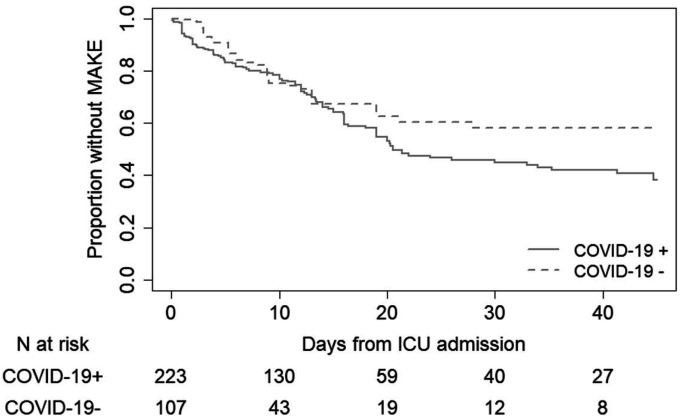
Association of COVID-19 status with major adverse kidney outcomes. Kaplan-Meier plot censored for hospital discharge. The Y-axis represents the proportion of patients free of the primary MAKE outcome. The X-axis represents time in the study. The solid line represents COVID-19 positive patients, and the dashed line indicates COVID-19 negative patients. The number of patients at-risk at each time is presented below the graph.

In the clinical study cohort, median hospital length of stay for the MAKE outcome was 10 days (IQR 5–19 days). The cumulative incidence of MAKE was 40% among COVID-19 patients (82 events) and 20% among negative controls (25 events; Figure 1). After inverse probability weighting by propensity-score and additional adjustment for baseline serum creatinine, SARS-CoV-2 positivity was associated with an estimated 70% greater incidence of MAKE (Table 2; relative risk 1.70; 95% CI 1.05–2.74; p-value = 0.03). SARS-CoV-2 positivity was associated with an estimated 7-times higher incidence of KRT (relative risk 7.41; 95% CI 1.69–32.41) and nearly 1.8-times higher incidence of death (relative risk 1.79; 95% CI 1.06–3.00). The associations of COVID-19 with MAKE were statistically similar after further adjusting for vasopressor use at study admission (**Supplemental Table 3**).

### Baseline markers of tubular injury, viability, and function

The biomarker cohort included 117 COVID-19 patients and 64 SARS-CoV-2 negative control patients (**Supplemental Table 1**). Patients in the biomarker cohort had modestly greater APACHE III and SOFA scores compared with those in the clinical cohort. The median urine albumin:creatinine ratio at baseline was 72.1 mg/g (IQR 24.7–143.7) in COVID-19 patients and 48.2 mg/g (IQR 21.9–197.9) in control patients. Nephrotic range proteinuria was present in only one patient, who was SARS-CoV-2 negative. COVID-19 negative patients tended to be on more home medications, although these differences were small after propensity score weighting (**Supplemental Table 2b**). Baseline urine concentrations of KIM-1 tended to be modestly lower, and the summary secretion score modestly higher, in COVID-19 positive compared with COVID negative patients (Table 3 and **Supplemental Table 2**). There was no association with COVID-19 status and baseline secretory solute urine:plasma ratios (**Supplemental Table 4**).

### Longitudinal changes in markers of tubular injury, viability, and function

**Figure 2:**
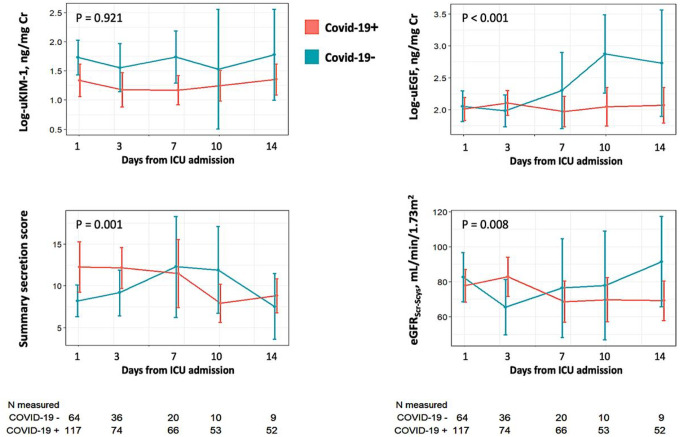
Longitudinal changes in markers of injury, viability, secretion, and estimated GFR. The Y-axes represent estimated GFR_creatinine+cystatin_, the summary tubular secretion score, and log urine concentrations of KIM-1 and EGF indexed to urine creatinine. The X-axes represent time in the study, with measurements performed on days 1. 3. 7, 10 and 14. The orange lines represent COVID-19 positive patients, and the green lines represent COVID-19 negative patients. Vertical bars represent 95% confidence intervals.

There was a median of three follow-up measurements in the biomarker cohort: 125 patients had at least two follow-up measurements, 93 had at least three measurements, and 61 had four or five measurements. After propensity-score inverse probability weighting and adjustment for informative censoring, urine KIM-1 concentrations remained significantly unchanged over time in both COVID-19 positive and COVID-19 negative patients. (Figure 2 and Table 4). In contrast, urine EGF concentrations increased by an average of 7% per day (95% CI 4.1% −10.0 per day) in the COVID-19 negative group but by only 0.5% per day (95% CI −1.1% to +2.2% per day) in COVID-19 positive group (p-value for interaction <0.001). Similar trends were observed for trajectories of the summary secretion score and estimated GFR, with modest increases over time in the COVID-19 negative group but negligible changes in the COVID-19 positive group. Individual secretory solute urine:plasma ratios tended to increase in COVID-19 negative patients and decrease in COVID-19 positive patients, with the most significant differences displayed by kynurenic acid and tiglylglycine (**Supplemental Figure 1**).

## DISCUSSION

Herein we have shown differential trajectories of markers of tubular injury, viability, and secretion between prospectively enrolled, critically ill patients with and without COVID-19. This study adds unique insight into the mechanisms of kidney injury in COVID-19 by illustrating patterns of tubular function over time in comparison with contemporaneously enrolled control persons without the disease. Among control patients, urine EGF concentrations, secretory clearance ratios, and eGFR increased over the course of the study, consistent with a pattern of kidney recovery. In contrast, these markers did not appreciably change in comparably ill patients with COVID-19. These findings suggest that COVID-19 may cause a more protracted and severe course of tubular dysfunction. Similar to other studies, we found COVID-19 to be associated with greater risks of kidney replacement therapy and death.

Proposed pathways of AKI in SARS-CoV-2 infection include a protracted inflammatory response, overstimulation of pro-thrombotic pathways, and direct viral infection of the kidneys.(24) A postmortem study found more extensive tubular necrosis and microvascular thrombosis in COVID-19 cases compared with bacterial sepsis.(25) Direct kidney infection of SARS-CoV-2 requires viremia, which is relatively uncommon and limited to severe cases of COVID-19, however more sensitive methods have detected SARS-CoV-2 in urine sediments suggesting kidney infection may be more common than previously appreciated.(12,13,26) Proximal tubule reabsorption defects have been reported in hospitalized patients with COVID-19, including phosphate loss, hypouricemia, and urine glucose wasting.(10,11) However, these studies lacked suitable control groups or longitudinal measures of function. We found that urine KIM-1 concentrations were similar between patients with and without COVID-19 over the course of this study, suggesting comparable tubular injury. Yet, patients without SARS-CoV-2 tended to recover eGFR and had a positive trend in EGF and the tubular secretory clearance compared to patients with COVID-19, suggesting a slower pattern of kidney recovery in COVID-19 which is consistent with clinical observation. (5,27)

Previous case series have reported relatively high incidences of AKI and KRT in critically ill patients with COVID-19. For example, the incidences of AKI and KRT were 51% and 19%, respectively, in a multicenter study of 3,309 persons with COVID-19 from ICUs across the United States.(7) Similarly high incidences of these outcomes have been reported in individual ICU-based studies of COVID-19.(28,29) In one of the few studies with a control group, the relative risk for AKI and KRT were 1.5 and 3.1, respectively, in 3,345 patients with COVID-19 and 1,265 patients without COVID-19 from the New York City area.(30) Another study comparing hospitalized patients with COVID-19 versus patients with a positive test for influenza found that COVID-19 was associated with a 2.1-times greater incidence of ≥ stage 2 AKI and a 53% lower chance of kidney recovery at discharge.(27) In the only prospective study, a single center in Switzerland enrolled 507 consecutive adults who presented with symptoms of respiratory infection. The incidence of AKI over the course of hospitalization was 2.5-times higher in patients who tested positive for SARS-CoV-2 compared to ßthose with another etiology of their respiratory illness.(31) Among these, our study is unique in focusing on critically ill persons with COVID-19 and comparing to a matched control group of patients with symptoms of a respiratory infection; in particular, we identified a substantially greater risk for KRT in COVID-19 compared to controls of comparable illness severity in the ICU.

Strengths of the current study include prospective enrollment of critically ill patients based on a clinical indication for SARS-CoV-2 testing and the use of propensity matching within the cohort to increase similarity between COVID-19 patients and negative controls. Longitudinal assessment of tubular injury, viability, and secretory clearance markers provides objective measures of these processes over the course of hospitalization. Several limitations of the study warrant comment. Despite matching on indication and propensity score, unmeasured differences between groups may have distorted associations with the trajectories of tubular markers and outcomes. We used statistical methods to account for differential dropout given the competing risk of death; nonetheless, unmeasured differences in surviving patients may have biased the observed associations. The selected markers of tubular injury, viability, and secretory clearance (KIM-1, EGF, and secretory solute ratios) may incompletely reflect these underlying biological processes. Individual secretory markers have differing affinities for tubular transporters, which in aggregate are intended to summarily reflect tubular secretion in absence of a true gold standard; the summary secretion score was created for ease of interpretation, although there may be a more optimal combination of markers. The calculation of eGFR while creatinine and cystatin C are not in steady-state may limit accuracy in monitoring kidney function trajectories. Finally, evolution of prevalent viral strains and practice patterns since the data collection period may limit generalizability.

In summary, we found SARS-CoV-2 infection to be associated with more severe AKI and a pattern of prolonged tubular dysfunction in comparably ill ICU patients with and without this infection.

## Figures and Tables

**Table 1.** Baseline characteristics of the clinical study cohort.

	Before propensity score weighting			After propensity score weighting		
	COVID-19 (N = 223)	No COVID-19 (N = 107)	Standardized mean difference	COVID-19 (N = 223)	No COVID-19 (N = 107)	Standardized mean difference
Age, years	54.8 ± 16.1	56.4 ± 17.1	0.10	55.4 ± 15.9	55.6 ± 18.4	0.01
Male	147 (66%)	66 (62%)	0.08	64%	58%	0.12
Race/ethnicity						
Asian	39 (17%)	7 (7%)		17%	6%	
Black	28 (13%)	19 (18%)		16%	16%	
White	130 (58%)	68 (64%)		56%	66%	
Other	26 (12%)	13 (12%)		11%	12%	
Baseline illness severity						
APACHE III	71.1 ± 28.2	77.4 ± 27.7	0.29	72.8 ± 21.6	70.9 ± 23.5	−0.08
SOFA	5.0 ± 4.4	6.0 ± 4.0	0.34	4.9 ± 3.0	5.3 ± 2.6	0.13
ARDS	87 (39%)	21 (20%)	0.42	33%	20%	0.29
Mechanical ventilation	104 (47%)	46 (43%)	0.08	45%	36%	0.18
Vasopressors	79 (35%)	37 (35%)	0.00	35%	22%	0.29
ECMO	16 (7%)	1 (1 %)	0.31	5%	3%	0.10
Serum creatinine, mg/dL	1.1 ± 0.7	1.1 ± 0.6	0.07	1.1 ± 0.6	1.0 ± 0.5	−0.12
Admission source						
Emergency room	82 (37%)	55 (51 %)		42%	46%	
Hospital transfer	105 (47%)	28 (26%)		40%	30%	
Inpatient	21 (9%)	5 (5%)		9%	9%	
Other source	0 (0%)	4 (4%)		0%	2%	
Body mass index, mg/kg^2^	31.2 ± 9.2	30.5 ± 10.8	−0.09	30.7 ± 8.3	29.8 ± 9.9	−0.10
Current tobacco use	21 (9%)	24 (22%)	0.37	10%	20%	0.28
Medical history						
Diabetes	71 (32%)	25 (23%)	0.20	29%	24%	0.11
Heart failure	29 (13%)	28 (26%)	0.33	16%	19%	0.08
COPD	14 (6%)	29 (27%)	0.59	11%	14%	0.09
Use of ACEi or ARB	61 (27%)	37 (35%)	0.17	31%	34%	0.06

APACHE = Acute Physiology, Age, Chronic Health Evaluation score; SOFA = Sequential Organ Failure Assessment score; ARDS = Acute Respiratory Distress Syndrome; ECMO = extracorporeal membrane oxygenation; COPD = chronic obstructive pulmonary disease, ACEi = angiotensin converting enzyme inhibitor, ARB = angiotensin receptor blocker. All values in the table are mean (SD) or number (%).

**Table 2.** Association of COVID-19 status with in-hospital kidney outcomes in the clinical cohort.

Outcome	Number at risk	Number of events	Adjusted relative risk^1^ (95% CI)	P-value
Major adverse kidney outcome^2^				
COVID-19 positive	223	98	1.70 (1.05, 2.74)	0.03
COVID-19 negative	107	25		
Acute kidney injury				
COVID-19 positive	223	93	1.34 (0.87, 2.08)	0.18
COVID-19 negative	107	30		
Doubling of serum creatinine				
COVID-19 positive	223	51	1.63 (0.75, 3.53)	0.22
COVID-19 negative	107	9		
Kidney replacement therapy				
COVID-19 positive	223	27	7.41 (1.69, 32.41)	0.008
COVID-19 negative	107	2		
Death				
COVID-19 positive	223	84	1.79 (1.06, 3.00)	0.03
COVID-19 negative	107	22		

1 Relative risk compares COVID-19 positive with COVID-19 negative patients using propensity-score inverse probability weighting and additional adjustment for baseline serum creatinine concentration.

2 Defined by at least a doubling of the serum creatinine concentration, kidney replacement therapy, or death.

**Table 3.** Baseline kidney measures by COVID-19 status in the biomarker cohort.

	Number of measurements	COVID-19 positive (N = 117)	COVID-19 negative (N = 64)	P-value^1^
eGFR-creatinine^2^	181	81.1 (53.2106.0)	84.5 (50.1–105.8)	0.60
eGFR-cystatin-C^2^	181	68.0 (31.7–102.8)	74.1 (36.9–98.4)	0.64
eGFR-creatinine-cystatin-C^2^	181	79.7 (39.0–109.7)	81.4 (45.1–94.6)	0.56
Urine ACR, mg/g	181	72.1 (24.7–143.7)	48.2 (21.9–197.9)	0.52
Urine ACR >3,000 mg/g, %	181	0%	1%	0.35
Serum glucose	140	151.0 (117.9–221.6)	133.0 (113.2–186.1)	0.22
Glucose wasting, %	140	9%	10%	0.81
				
Urine KIM-1, ng/mg Cr	181	4.4 (2.3–9.0)	4.7 (3.2–10.9)	0.06
Urine EGF, ng/mg Cr	181	7.3 (4.4–12.8)	7.8 (4.0–12.4)	0.79
Summary secretion score	180	9.4 (4.6–16.6)	6.1 (3.1–11.0)	0.07

1 P-values test the equality of weighted geometric means for continuous variables and the equality of weighted proportions for categorical variables.

2 CKD-EPI2021 equation, ml/min/1.73m^2^.

**Table 4.** Estimated trajectories of kidney markers in critically ill adults with and without COVID-19

Marker	Mean percent change per day (95% CI)*		P-value for interaction
	COVID-19 positive	COVID-19 negative	
**KIM-1**	0.4 (−1.9, 2.7)	0.0 (−6.1, 6.6)	0.92
**EGF**	0.5 (−1.1, 2.2)	7.0 (4.1, 10.0)	0.0002
**Summary secretion score**	−0.3 (−0.5, 0.0)	0.3 (0.1, 0.6)	0.001

	Mean ml/min/1.73m^2^ change per day (95% CI)*		
	COVID-19 positive	COVID-19 negative	
**eGFR**_**creatinine**_	0.2 (−0.4, 0.8)	2.1 (1.2, 3.0)	0.0008
**eGFR**_**cystatin**_	−1.0 (−1.7, −0.3)	0.3 (−0.8, 1.4)	0.045
**eGFR**_**creatinine-cystatin**_	−0.6 (−1.3, 0.0)	1.1 (0.0, 2.1)	0.008

	Mean change per day (95% CI)*		
Individual secretory solute Urine:plasma ratio	COVID-19 positive	COVID-19 negative	
Cinnamoylglycine	−2.2 (−3.8, −0.6)	3.9 (−1.2, 9.0)	0.02
Indoxyl sulfate	−1.9 (−6.3, 2.6)	2.3 (−2.9, 7.4)	0.26
Isovalerylglycine	−15.0 (−30.4, 0.3)	14.5 (−6.7, 35.7)	0.03
Kynurenic acid	−3.9 (−8.7, 0.8)	8.2 (2.9, 13.5)	0.0008
P-cresol sulfate	−0.2 (−2.2, 1.8)	0.6 (−1.2, 2.3)	0.60
Pyridoxic acid	−20.4 (−41.6, 0.7)	23.7 (0.6, 46.7)	0.005
Tiglylglycine	−7.5 (−14.5, −0.5)	8.1 (2.3, 14.0)	0.0008
Xanthosine	−3.3 (−5.6, −1.1)	0.5 (−6.9, 7.8)	0.33

* After propensity-score inverse probability weighting and adjustment for informative censoring.

## Data Availability

The datasets generated during and/or analyzed during the current study are not publicly available currently due to ongoing research studies, but the data are available from the corresponding author on reasonable request.

## References

[1] Oran DP, Topol EJ. The Proportion of SARS-CoV-2 Infections That Are Asymptomatic: A Systematic Review. Ann Intern Med May. 2021;174(5):655–62. 10.7326/M20-6976 PMC7839426 33481642

[2] Stokes EK, Zambrano LD, Anderson KN. Coronavirus Disease 2019 Case Surveillance - United States. Jun. 2020;19 2020;69(24):759–765. 10.15585/mmwr.mm6924e2 PMC7302472 32555134

[3] Helms L, Marchiano S, Stanaway IB. Cross-validation of SARS-CoV-2 responses in kidney organoids and clinical populations. JCI Insight Dec. 2021;6(24). 10.1172/jci.insight.154882 PMC8783682 34767537

[4] Chung JJ, Goldstein L, Chen YJ. Single-Cell Transcriptome Profiling of the Kidney Glomerulus Identifies Key Cell Types and Reactions to Injury. J Am Soc Nephrol Oct. 2020;31(10):2341–54. 10.1681/ASN.2020020220 PMC7609001 32651223

[5] Chan L, Chaudhary K, Saha A, Chauhan K, Vaid A, Zhao S, AKI in Hospitalized Patients with COVID-19. JASN. 2021;32(1):151–60. 32883700 10.1681/ASN.2020050615 PMC7894657

[6] Hirsch JS, Ng JH, Ross DW. Acute kidney injury in patients hospitalized with COVID-19. Kidney Int Jul. 2020;98(1):209–18. 10.1016/j.kint.2020.05.006 PMC7229463 32416116

[7] Gupta S, Coca SG, Chan L, Melamed ML, Brenner SK, Hayek SS, AKI Treated with Renal Replacement Therapy in Critically Ill Patients with COVID-19. JASN. 2021;32(1):161–76. 33067383 10.1681/ASN.2020060897 PMC7894677

[8] Chen YT, Shao SC, Hsu CK, Wu IW, Hung MJ, Chen YC. Incidence of acute kidney injury in COVID-19 infection: a systematic review and meta-analysis. Crit Care Jun. 2020;24(1). 10.1186/s13054-020-03009-y PMC7296284 32546191

[9] Menez S, Moledina DG, Thiessen-Philbrook H, Wilson FP Obeid W, Simonov M, Prognostic Significance of Urinary Biomarkers in Patients Hospitalized With COVID-19. Am J Kidney Dis. 2022;79(2):257–267.e1. 34710516 10.1053/j.ajkd.2021.09.008 PMC8542781

[10] Kormann R, Jacquot A, Alla A. Coronavirus disease 2019: acute Fanconi syndrome precedes acute kidney injury. Clin Kidney J Jun. 2020;13(3):362–70. 32695327 10.1093/ckj/sfaa109 PMC7314200

[11] Werion A, Belkhir L, Perrot M. SARS-CoV-2 causes a specific dysfunction of the kidney proximal tubule. Kidney Int Nov. 2020;98(5):1296–307. 32791255 10.1016/j.kint.2020.07.019 PMC7416689

[12] L CD E RV, ND ZC. Relevant SARS-CoV-2 viremia is associated with COVID-19 severity: Prospective cohort study and validation cohort. J Med Virol Nov. 2022;94(11):5260–70. 35811284 10.1002/jmv.27989 PMC9349374

[13] Roshandel MR, Nateqi M, Lak R. Diagnostic and methodological evaluation of studies on the urinary shedding of SARS-CoV-2, compared to stool and serum: A systematic review and meta-analysis. Cell Mol Biol (Noisy-le-grand) Sep. 2020;66(6):148–56. 33040802

[14] Bhatraju PK, Morrell ED, Stanaway IB. Angiopoietin-Like4 Is a Novel Marker of COVID-19 Severity. Crit Care Explor Jan. 2023;5(1). 10.1097/CCE.0000000000000827 PMC9803343 36600780

[15] Vincent JL, Moreno R, Takala J. The SOFA (Sepsis-related Organ Failure Assessment) score to describe organ dysfunction/failure. On behalf of the Working Group on Sepsis-Related Problems of the European Society of Intensive Care Medicine Intensive Care Med Jul. 1996;22(7):707–10. 10.1007/BF01709751 8844239

[16] Brown SM, Peltan ID, Barkauskas C. What Does Acute Respiratory Distress Syndrome Mean during the COVID-19 Pandemic? Ann Am Thorac Soc. Dec. 2021;18(12):1948–50. 34288834 10.1513/AnnalsATS.202105-534PS PMC8641820

[17] McKown AC, Wang L, Wanderer JP Predicting Major Adverse Kidney Events among Critically Ill Adults Using the Electronic Health Record. J Med Syst Aug. 2017;41(10). 10.1007/s10916-017-0806-4 PMC5821255 28861688

[18] Disease K. Improving Global Outcomes (KDIGO) Acute Kidney Injury Work Group. KDIGO Clinical Practice Guideline for Acute Kidney Injury. Kidney inter. 2012;2:1–138.

[19] Inker LA, Eneanya ND, Coresh J, Tighiouart H, Wang D, Sang Y, New Creatinine- and Cystatin C-Based Equations to Estimate GFR without Race. New England Journal of Medicine. 2021;385(19):1737–49. 34554658 10.1056/NEJMoa2102953 PMC8822996

[20] Granda ML, Zelnick LR, Prince DK, Hoofnagle A, Young BA, Kestenbaum BR. Tubular Secretion and Estimated GFR Decline in the Jackson Heart Study. Kidney International Reports [Internet]. 2022 Sep 12 [cited 2022 Oct 12]; Available from: https://www.sciencedirect.com/science/article/pii/S2468024922017697 10.1016/j.ekir.2022.09.008 PMC9727527 36506244

[21] Garimella PS, Katz R, Waikar SS, Srivastava A, Schmidt I, Hoofnagle A, Kidney Tubulointerstitial Fibrosis and Tubular Secretion. American Journal of Kidney Diseases. 2022;79(5):709–16. 34571064 10.1053/j.ajkd.2021.08.015 PMC8973399

[22] Stuart EA. Matching methods for causal inference: A review and a look forward. Stat Sci Feb. 2010;25(1):1–21. 20871802 10.1214/09-STS313 PMC2943670

[23] Zeger SL, Liang KY. Longitudinal data analysis for discrete and continuous outcomes. Biometrics Mar. 1986;42(1):121–30. 3719049

[24] Nadim MK, Forni LG, Mehta RL. COVID-19-associated acute kidney injury: consensus report of the 25th Acute Disease Quality Initiative (ADQI) Workgroup. Nat Rev Nephrol Dec. 2020;16(12):747–64. 33060844 10.1038/s41581-020-00356-5 PMC7561246

[25] Volbeda M, Jou-Valencia D, Heuvel MC. Comparison of renal histopathology and gene expression profiles between severe COVID-19 and bacterial sepsis in critically ill patients. Crit Care Jun. 2021;25(1). 10.1186/s13054-021-03631-4 PMC8190989 34112226

[26] Caceres PS, Savickas G, Murray SL. High SARS-CoV-2 Viral Load in Urine Sediment Correlates with Acute Kidney Injury and Poor COVID-19 Outcome. J Am Soc Nephrol Oct. 2021;32(10):2517–28. 10.1681/ASN.2021010059 PMC8722807 34088853

[27] Strohbehn IA, Zhao S, Seethapathy H. Acute Kidney Injury Incidence, Recovery, and Long-term Kidney Outcomes Among Hospitalized Patients With COVID-19 and Influenza. Kidney Int Rep Oct. 2021;6(10):2565–74. 34307971 10.1016/j.ekir.2021.07.008 PMC8280679

[28] Argenziano MG, Bruce SL, Slater CL. Characterization and clinical course of 1000 patients with coronavirus disease 2019 in New York: retrospective case series. BMJ May. 2020;29 369:m1996. 10.1136/bmj.m1996 PMC7256651 32471884

[29] Azoulay E, Fartoukh M, Darmon M. Increased mortality in patients with severe SARS-CoV-2 infection admitted within seven days of disease onset. Intensive Care Med Sep. 2020;46(9):1714–22. 32780165 10.1007/s00134-020-06202-3 PMC7417780

[30] Fisher M, Neugarten J, Bellin E. AKI in Hospitalized Patients with and without COVID-19: A Comparison Study. J Am Soc Nephrol Sep. 2020;31(9):2145–57. 10.1681/ASN.2020040509 PMC7461660 32669322

[31] Diebold M, Zimmermann T, Dickenmann M. Comparison of Acute Kidney Injury in Patients with COVID-19 and Other Respiratory Infections: A Prospective Cohort Study. J Clin Med May. 2021;10(11). 10.3390/jcm10112288 PMC8197451 34070339

